# An outbreak of classical swine fever in pigs in Bangladesh, 2015

**DOI:** 10.1002/vms3.81

**Published:** 2017-11-20

**Authors:** Shamim Sarkar, Mohammad Enayet Hossain, Emily S. Gurley, Rashedul Hasan, Mohammed Z. Rahman

**Affiliations:** ^1^ Infectious Diseases Division Programme for Emerging Infections icddr,b Dhaka Bangladesh

**Keywords:** Bangladesh, classical swine fever, outbreak, pig

## Abstract

In a group of 22 healthy pigs aged between 4 and 6 months, 2 pigs became ill with high fever, complete anorexia, cough and abnormal swaying movements on 22 June 2015. One of them died on June 24 and the second died on July 3. Shortly after, the remaining pigs also fell ill and died from the same illness by 10 August 2015. We investigated the aetiology, epidemiological and clinical features of the outbreak. We recorded the clinical signs and symptoms for each pig with the date of onset of illness. Veterinarians conducted *post‐mortem* examinations on the 12 dead pigs, they collected tissue samples from the dead pigs and placed them in a tube containing 1 mL of nucleic acid extraction buffer (lysis buffer). We tested all the tissue samples by real‐time reverse transcription polymerase chain reaction (rRT‐PCR) to detect classical swine fever virus (CSFV) because the animals’ symptoms matched those of this disease. We also conducted a phylogentic analysis of the nucleotide sequence of the E2 gene segment of CSFV detected in a lung tissue sample. The attack rate (22/22) and the case fatality were 100%. The predominant symptoms of the disease included high fever, cough, diarrhoea and swaying movements of the hind legs prior to death. Of the 12 pigs tissue samples tested, all had evidence of the presence of CSFV RNA by rRT‐PCR. The phylogenetic analysis indicated that the virus belongs to genotype 2.2, which is closely related to CSFV genotype 2.2 reported in India. Our investigation suggests that CSF is circulating in pigs, posing a risk for communities in Bangladesh that rely on pigs for economic income and dietary protein. Future research could focus on estimating the disease and economic burden of CSFV in pig rearing areas to determine if interventions might be warranted or cost‐effective.

## Introduction

Classical swine fever virus (CSFV), a member of the *Pestivirus* genus within the Flaviviridae family (Meyers *et al*. 1989), is a highly contagious and often fatal infection in pigs which contributes to economic loss in pig farming (Edwards *et al*. 2000; Moennig *et al*. 2003; Postel *et al*. 2012) by constraining pig production and diminishing international trade in regions with CSFV‐infected pigs and pig products (Boender *et al*. 2008; Sarma *et al*. 2008). CSFV has three major genotypes: 1, 2 and 3, and each genotype is categorized into three to four subtypes (Paton *et al*. 2000).

CSF has been reported in most Indian states (first being reported in 1962; Patil *et al*. 2010), and in Nepal (Jha *et al*. 2012) and Bhutan (Monger 2015), which have also reported sporadic CSF outbreaks in their countries.

CSF has been controlled and eradicated from most of the countries using vaccination campaigns (Van Oirschot 2003; Greiser‐Wilke & Moennig 2004; Song *et al*. 2013; Luo *et al*. 2014). However, CSF remains a threat to pigs raised in South Asia, including India, Nepal and Bhutan (Gatenby & Chemjong 1992; Dukpa *et al*. 2011; Prasad *et al*. 2011).

India has CSF prevention and control measures in place including available locally produced CSF vaccines (Bett *et al*. 2012), Bhutan has CSF vaccination programs for government breeding farms only (Monger 2015) and Nepal has planned to implement the national classical swine fever control program (Animal Health, Kathmandu, Nepal). In Bangladesh, there is an absence of official CSF control measures, including CSF vaccination (Department of Livestock Service, Bangladesh).

CSF is a notifiable disease in India, Nepal and Bhutan (Monger 2015) and Bangladesh (Department of Livestock Services, Bangladesh).

Pig production in Bangladesh is a highly decentralized production system, coupled with stigmatization (Nahar *et al*. 2012b), combined to make surveillance of swine diseases difficult. Although Bangladesh is a predominantly Muslim country, there are minority ethnic communities that raise non‐descriptive indigenous types of pigs (Nahar *et al*. 2012b). In 2009, the Department of Livestock Services (DLS) estimated that there were ~200 000 pigs in the country (DLS, unpublished data). A study found that 88% of pigs were raised in a backyard farm (Khan *et al*. 2014). Most of the pig raisers were impoverished and invested minimally in pig feed, housing and preventive care (Nahar *et al*. 2012a,b). However, pig rearing is an important source of livelihood and animal protein for these communities in Bangladesh (Nahar *et al*. 2015).

## Case report setting

**Figure 1:**
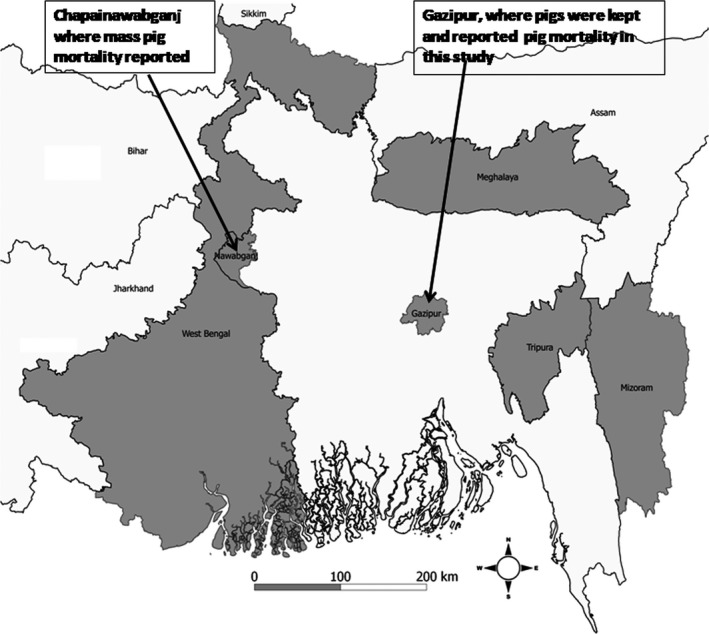
Map showing the area where pigs were kept, where mass pigs mortality occurred and various states (sharing borders with Bangladesh) of India which have reported classical swine fever (CSF) outbreak (shaded in grey).

In 2015, on day zero, we purchased 22 seemingly healthy pigs aged between 4 and 6 months from the northwestern Noagaon district to study the immunogenicity of the live attenuated Japanese encephalitis (JE) vaccine. On day 2, we moved the pigs to Gazipur, located in central Bangladesh (Fig. 1). We chose Gazipur because this is an urban area where JE is unlikely to circulate. Trained veterinarians administered one dose of the JE vaccine on day 5 by intramuscular injection. On the same day, prior to administering the vaccine, all pigs were bled to look for pre‐existing antibodies to JE.

## Case report

All pigs were kept in one confinement pen under continuous monitoring (observed 4 times a day) by a trained research assistant. Once the pigs showed illness (fever, cough or diarrhoea), the research assistant informed the study veterinarian about this illness, the study veterinarian then performed a clinical examination and the sick pigs were transferred to an isolation pen and provided with symptomatic and supportive treatment to those pigs.

Two pigs became ill with high fever, complete anorexia, cough and abnormal swaying movements the day after their vaccinations (on June 22). One died on June 24 and the second died on July 5. The pigs continued to fall ill and die due to the illness despite veterinarians treating the animals for their signs. Following the death of the first pig and with the continuing illness in the other pigs (e.g. high fever, cough, diarrhoea, complete anorexia, reluctance to move, shivering and swaying movements in the hind quarter for 2–10 days prior to their death), we suspected that the pigs might have contracted CSF infections. We investigated to confirm the causative agent and describe the epidemiological and clinical features of the outbreak.

### *Post‐mortem* examination

Trained veterinarians recorded the clinical signs of each pig with dates of onset of illness. To determine the clinical–pathological features, the team also conducted *post‐mortem* examinations on 12 dead pigs (>50%); they collected tissue samples from the liver, kidneys, lungs, spleen, tonsils and brain of the pigs and placed each tissue sample in a tube containing 1 mL of nucleic acid extraction buffer (lysis buffer).

### Laboratory investigations

We tested all the tissue samples by real‐time one‐step reverse transcription polymerase chain reaction (rRT‐PCR) to detect CSFV (Dias *et al*. 2014).

All the pigs belonged to a single herd and were grouped together in one pen, and so were most likely infected with the same pathogen. Therefore, one randomly selected sample was subjected to sequence based analysis for CSFV genotyping. We prepared cDNA and subsequently performed PCR to amplify a 1342‐bp segment of the E2 gene of CSFV. We sequenced the PCR product using Sanger's sequencing method (Jiang *et al*. 2013). We constructed a phylogenetic tree which included the E2 sequence of our porcine strain (accession no.: KX345847) along with the related global strains deposited in the GenBank database by maximum likelihood reconstruction of sequence alignments using Molecular Evolutionary Genetics Analysis (MEGA) 6. We also calculated frequency, percentage and mean for the variables related to pig demographic characteristics and clinical manifestation.

## Results

**Figure 2:**
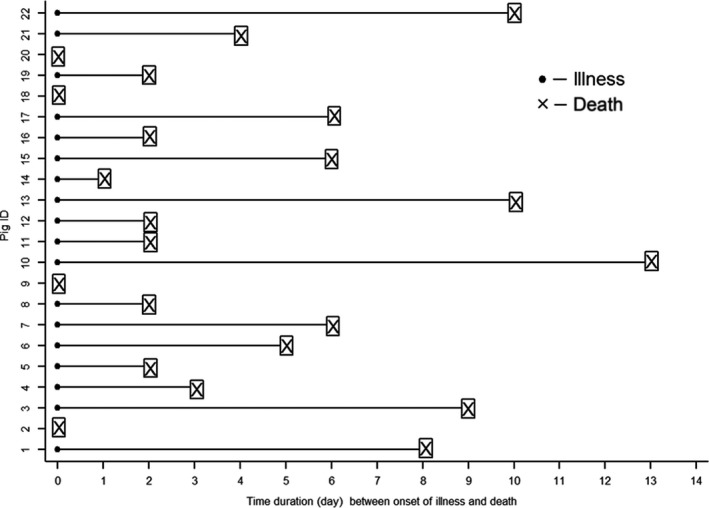
Time duration between onset of illness and death of pigs (*n* = 22).

**Figure 3:**
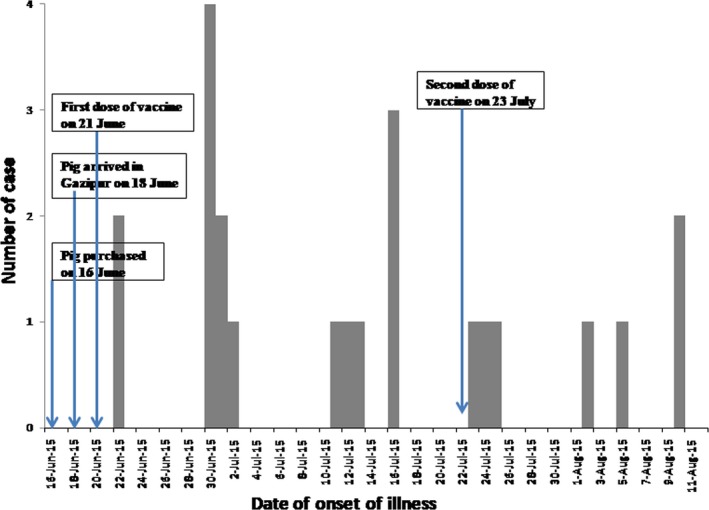
Temporal distribution of date of onset of illness of classical swine fever (CSF) among pigs during this outbreak in Bangladesh, June–August 2015.

**Figure 4:**
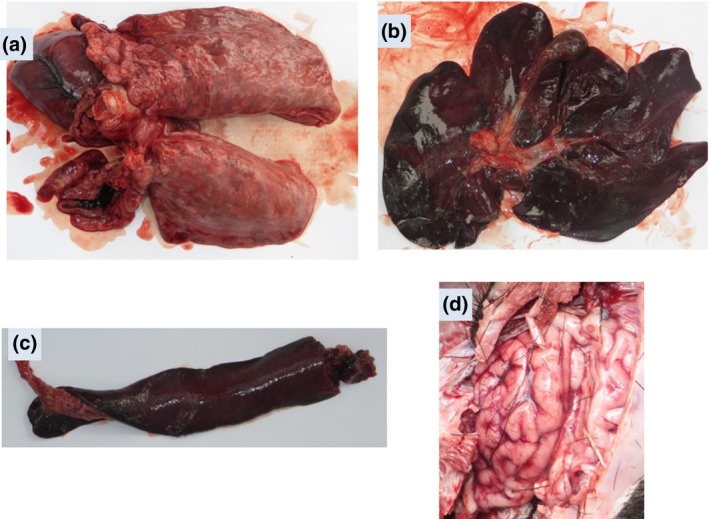
Classical swine fever (CSF)‐related lesions: (a) congested lungs, (b) haemorrhagic and dark tan‐coloured liver, (c) haemorrhagic and dark tan‐coloured spleen and (d) slightly haemorrhagic brain.

The mean age of the pigs was 4.5 months and their mean weight was 11.2 kg. The majority (55%) of the pigs were male. The infection rate (22/22) and the case fatality rate were 100%. The mean duration between illness onset and death was 4 days (range 0–13 days) (Fig. 2). Predominant signs included high fever (82%), cough (50%), diarrhoea (50%), huddling (41%) and swaying movements of the hind quarter (36%) prior to their death (Table 1). All pigs were dead by day 65 (Fig. 3). The gross changes recorded included congested lungs, a haemorrhagic and dark tan‐coloured liver, focal haemorrhage, a black/tan‐coloured spleen, a slightly haemorrhagic brain, enlarged tonsils and pinpoint haemorrhaging in the kidneys (Fig. 4). All 12 pig tissues samples tested positive for CSFV RNA by rRT‐PCR.

**Table 1:** Demographic and clinical characteristics of CSF‐infected pigs in Bangladesh, 2015 (*n* = 22)

Variables	
Age (months), mean (SD)	4.5 (0.47)
Body weight (kg), mean (SD)	11.2 (1.97)
Sex, *n* (%)	
Male	12 (55)
Clinical manifestation, *n* (%)	
Fever	18 (82)
Cough	11 (50)
Diarrhoea	11 (50)
Complete anorexia	9 (41)
Huddling	9 (41)
Reluctance to move	9 (41)
Shivering	8 (36)
Swaying movement	8 (36)

**Figure 5:**
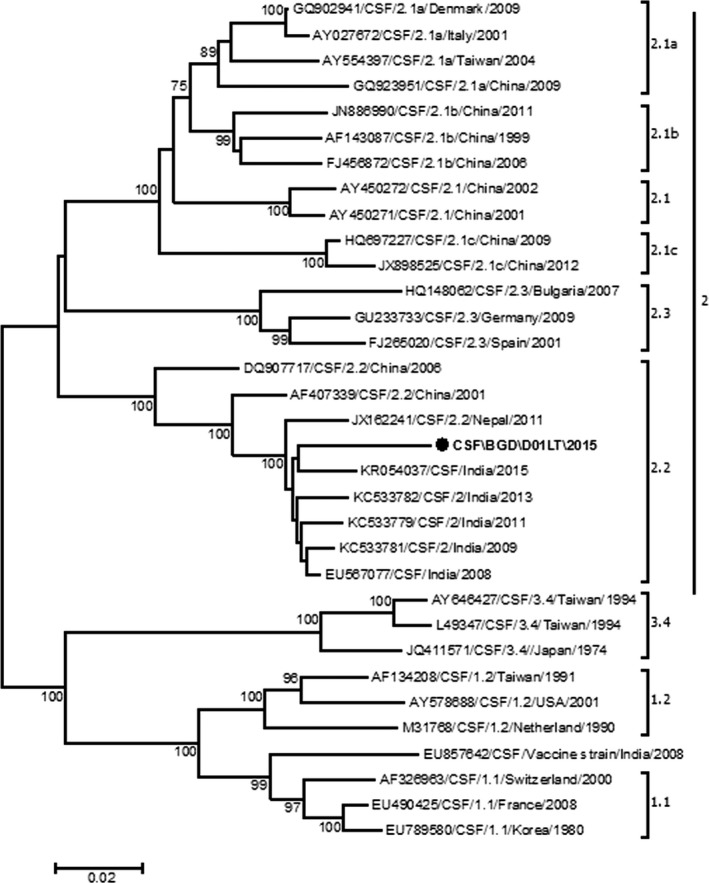
Phylogenetic tree of partial nucleotide sequences (1343 bp) of classical swine fever virus (CSFV) E2 gene. Numbers indicate the bootstrap values (100 replicates), and only the values above 70% are shown in the figure. Horizontal distances are proportional to sequence distances. The circle indicates Bangladesh CSFV strain.

We were able to amplify the E2 (1343 bp region) gene segment of a CSF positive lung tissue sample. The phylogenetic analysis indicates that it belongs to genotype 2.2 CSF virus, which is closely related to CSFV genotype 2.2 in India (95% at nucleotide level) (Fig. 5).

## Discussion

The clinical, necropsy and molecular findings suggested that the pigs had the CSFV infection. We identified a high fatality rate in CSF‐infected pigs which is comparable to other studies (Laevens *et al*. 1999; Dewulf *et al*. 2000). In this outbreak, all pigs showed clinical signs comparable to other studies of CSFV (Moennig *et al*. 2003; Malmarugan *et al*. 2014). Moreover, the necropsy findings of this study are similar to the findings from other pigs that died of CSFV infection in India (Ravishankar *et al*. 2007; Malmarugan *et al*. 2014). To the best of our knowledge, this is the first report of CSF‐associated pig mortality in Bangladesh. However, CSF‐associated pig mortality has already been reported in India, Nepal and Bhutan (Jha *et al*. 2012; Monger 2015; Khatoon *et al*. 2017).

We did not rule out the exact source of introduction of CSF in this study. However, we assume that the outbreak of CSF is likely to be due to introduction from the pig herd where we purchased these pigs. The pigs herd could be infected with CSF asymptomatically during purchasing of these pigs. We followed up with the pig herd owner over the phone about the signs and mortalities in their pig herd. They reported a similar illness (predominant signs were fever, cough, diarrhoea and reluctance to move prior to death) had occurred in their herds, indicating that these pig herds may have been exposed to CSFV. Moreover, a mass mortality of pigs raised in backyards in northwestern Bangladesh (Fig. 1) which is close to the border of India, with similar signs, was also reported in the year leading up to this event (Hasan Ali, personal communication). In addition, our CSFV sequence data revealed that the Bangladeshi strain of CSFV belongs to genotype 2.2. The strain exhibited highest identity (95% at nucleotide level) and a tight phylogenetic affiliation (Fig. 5) with the Indian strains of CSF genotype 2.2 found during 2008–2015 (Desai *et al*. 2010; Patil *et al*. 2010; Sarma *et al*. 2011). These findings also suggest that the source of introduction of CSF in this study could be the pig herd where we purchased our study pigs.

The onset of illness in two pigs was the day following vaccination (Fig. 3). The incubation period for CSFV is 2–14 days (Blome *et al*. 2017), which also suggests that these pigs were infected before they received the vaccine. In addition, we gave this same vaccine to 971 of pigs in the area on 27 June 2015, and did not receive any reports of illness in vaccinated pigs suggesting that the vaccine was not contaminated. These indicate that outbreaks were not caused by vaccination, but rather due to exposure prior to purchase.

Due to the lack of veterinary care, surveillance and diagnostic capacity in pig raising communities in Bangladesh (Nahar *et al*. 2012b), many outbreaks of CSF in pigs remain undiagnosed or underreported.

India uses locally produced CSFV vaccines as CSF prevention and control measures in northeastern states of India (Bett *et al*. 2012). Bhutan uses locally produced CSFV vaccine in government pig breeding farms only (Monger 2015). Nepal has planned to implement the national CSF control program (Animal Health, Kathmandu, Nepal). Bangladesh is now exploring CSF surveillance and control options now that there are reports of its circulation.

Securing resources and support for prioritizing efforts to control CSF in Bangladesh will depend on compelling data about the disease, the economic burden of CSF and future research should include estimating this burden. Effective interventions have been implemented in India (Bett *et al*. 2012), which may be culturally and economically appropriate for Bangladesh if the disease burden justifies intervention.

Our study had limitations. We did not test the pre‐vaccination serum samples of pigs for detecting CSF status (antibodies against CSFV). However, the finding of this study associated with unexpected event in accordance with our protocol. Moreover, the serum samples are no more available to do further analysis. Therefore, testing further for CSFV antibodies is beyond the scope of the study. In addition, all pigs were younger (mean age was 4.5 months) in this event, and we assume that low seroprevalence rate of CSFV in young pigs as it is supported by studies led by Kaden *et al*. (2000, 2002). Moreover, our virological findings indicate that the pigs could be non‐immune to CSFV; therefore, they were infected with CSFV as we detected CSFV RNA in their tissue samples.

### Nucleotide sequence accession number

The nucleotide sequences of CSF\BGD\DO1LT\2015 strain was deposited in GenBank under accession number KX345847.

## Source of funding

The study received support from the Grand Challenges Canada (grant no: 0506‐01‐10).

## Conflict of interest

The authors declare that they have no conflicts of interest.

## Contribution

Study design: SS, Sample testing: MEH, RH, MZR, Statistical analysis: SS, Manuscript draft: SS Revision and manuscript approval: SS, MEH, ESG, RH, MZR.

## Ethics statement

The authors confirm that the ethical policies of the journal, as noted on the journal's author guidelines page, have been adhered to and the appropriate ethical review committee approval has been received. The US National Research Council's guidelines for the Care and Use of Laboratory Animals were followed.

## References

[vms381-bib-0002] Bett B. , Deka R. , Padmakumar V. , Rajasekhar M (2012). Classical swine fever in northeast India: Prevention and control measures. ILRI Policy Brief 7. ILRI: Nairobi, Kenya.

[vms381-bib-0502] Blome S. , Staubach C. , Henke J. , Carlson J. & Beer M. (2017) Classical Swine Fever—An Updated Review. Viruses 9, 86. 10.3390/v9040086 PMC5408692 28430168

[vms381-bib-0003] Boender G.J. , Nodelijk G. , Hagenaars T.J. , Elbers A.R. & de Jong M.C. (2008) Local spread of classical swine fever upon virus introduction into The Netherlands: Mapping of areas at high risk. BMC veterinary research 4, 1. 18298803 10.1186/1746-6148-4-9 PMC2311289

[vms381-bib-0004] Desai G. , Sharma A. , Kataria R. , Barman N. & Tiwari A. (2010) 5′‐UTR‐based phylogenetic analysis of classical swine fever virus isolates from India. Acta Virologica 54, 79–82. 20201618 10.4149/av_2010_01_79

[vms381-bib-0005] Dewulf J. , Laevens H. , Koenen F. , Vanderhallen H. , Mintiens K. , Deluyker H. *et al* (2000) An experimental infection with classical swine fever in E2 sub‐unit marker‐vaccine vaccinated and in non‐vaccinated pigs. Vaccine 19, 475–482. 11027811 10.1016/s0264-410x(00)00189-4

[vms381-bib-0006] Dias N.L. , Fonseca Júnior A.A. , Oliveira A.M. , Sales É.B. , Alves B.R.C. & Dorella F.A. (2014) Validation of a Real Time PCR for Classical Swine Fever Diagnosis. Veterinary Medicine International 17, 4;Article ID 171235. 10.1155/2014/171235 PMC4000670 24818039

[vms381-bib-0007] Dukpa K. , Robertson I.D. & Ellis T.M. (2011) The seroprevalence of foot‐and‐mouth disease in the sedentary livestock herds in four districts of Bhutan. Preventive Veterinary Medicine 100, 231–236. 21570142 10.1016/j.prevetmed.2011.04.013

[vms381-bib-0008] Edwards S. , Fukusho A. , Lefevre P.‐C. , Lipowski A. , Pejsak Z. , Roehe P. *et al* (2000) Classical swine fever: The global situation. Veterinary microbiology 73, 103–119. 10785321 10.1016/s0378-1135(00)00138-3

[vms381-bib-0009] Gatenby R.M. & Chemjong P.B. (1992) Reproduction of pigs in the hills of eastern Nepal. Tropical animal health and production 24, 135–142. 1304659 10.1007/BF02359602

[vms381-bib-0010] Greiser‐Wilke I. & Moennig V. (2004) Vaccination against classical swine fever virus: Limitations and new strategies. Animal Health Research Reviews 5, 223–226. 15984328 10.1079/ahr200472

[vms381-bib-0011] Jha V.C. , Karna V.K. & Singh D.K. (2012) Classical swine fever in Nepal. Veterinary Record 171, 24. 22772845 10.1136/vr.e4594

[vms381-bib-0012] Jiang D.‐L. , Gong W.‐J. , Li R.‐C. , Liu G.‐H. , Hu Y.‐F. , Ge M. *et al* (2013) Phylogenetic analysis using E2 gene of classical swine fever virus reveals a new subgenotype in China. Infection, Genetics and Evolution 17, 231–238. 10.1016/j.meegid.2013.04.004 23608662

[vms381-bib-0500] Kaden V. , Lange E. , Fischer U. , & Strebelow G. (2000) Oral immunisation of wild boar against classical swine fever: evaluation of the first field study in Germany. Veterinary microbiology 73, 239–252. 10785331 10.1016/s0378-1135(00)00148-6

[vms381-bib-0501] Kaden V. , Heyne H. , Kiupel H. , Letz W. , Kern B. , Lemmer U. , Gossger K. , Rothe A. , Böhme H. , & Tyrpe P. (2002) Oral immunisation of wild boar against classical swine fever: concluding analysis of the recent field trials in Germany. Berliner und Munchener Tierarztliche Wochenschrift 115(5–6), 179–185. 12058591

[vms381-bib-0013] Khan S.U. , Salje H. , Hannan A. , Islam M.A. , Bhuyan A.M. , Islam M.A. *et al* (2014) Dynamics of Japanese encephalitis virus transmission among pigs in Northwest Bangladesh and the potential impact of pig vaccination. PLoS Neglected Tropical Diseases 8, e3166. 25255286 10.1371/journal.pntd.0003166 PMC4177832

[vms381-bib-0014] Khatoon E. , Barman N.N. , Deka M. , Rajbongshi G. , Baruah K. , Deka N. *et al* (2017) Molecular characterization of classical swine fever virus isolates from India during 2012–14. Acta Tropica 170, 184–189. 28279702 10.1016/j.actatropica.2017.03.004

[vms381-bib-0015] Laevens H. , Koenen F. , Deluyker H. & De Kruif A. (1999) Experimental infection of slaughter pigs with classical swine fever virus: Transmission of the virus, course of the. The veterinary record 145, 248. 10504066 10.1136/vr.145.9.243

[vms381-bib-0016] Luo Y. , Li S. , Sun Y. & Qiu H.J. (2014) Classical swine fever in China: A minireview. Veterinary microbiology 172, 1–6. 24793098 10.1016/j.vetmic.2014.04.004

[vms381-bib-0017] Malmarugan S. , Sundaram A.M. & Rajeswar J.J. (2014) An Outbreak of Classical Swine Fever in Indigenous Pigs in Tamil Nadu, India. International Journal of Advanced Veterinary Science and Technology 3, 145–148.

[vms381-bib-0018] Meyers G. , Rümenapf T. & Thiel H.‐J. (1989) Molecular cloning and nucleotide sequence of the genome of hog cholera virus. Virology 171, 555–567. 2763466 10.1016/0042-6822(89)90625-9

[vms381-bib-0019] Moennig V. , Floegel‐Niesmann G. & Greiser‐Wilke I. (2003) Clinical signs and epidemiology of classical swine fever: A review of new knowledge. The Veterinary Journal 165, 11–20. 12618065 10.1016/s1090-0233(02)00112-0

[vms381-bib-0020] Monger V.R. (2015). Epidemiology of selected pig viral diseases in Bhutan. Doctoral dissertation, Utrecht University.

[vms381-bib-0021] Nahar N. , Uddin M. , Gurley E.S. , Khan M.S.U. , Hossain M.J. , Sultana R. *et al* (2012a) Pig illnesses and epidemics: A qualitative study on perceptions and practices of pig raisers in Bangladesh. Veterinaria Italiana 48, 157–165. 22718332

[vms381-bib-0022] Nahar N. , Uddin M. , Sarkar R.A. , Gurley E.S. , Uddin K.M. , Hossain M.J. *et al* (2012b) Exploring pig raising in Bangladesh: Implications for public health interventions. Veterinaria Italiana 49, 7–17. 23564585

[vms381-bib-0023] Nahar N. , Uddin M. , Gurley E.S. , Hossain M.J. , Sultana R. & Luby S.P. (2015) Cultural and Economic Motivation of Pig Raising Practices in Bangladesh. EcoHealth 12, 611–620. 26122206 10.1007/s10393-015-1046-z PMC4696915

[vms381-bib-0024] Patil S. , Hemadri D. , Shankar B. , Raghavendra A. , Veeresh H. , Sindhoora B. *et al* (2010) Genetic typing of recent classical swine fever isolates from India. Veterinary microbiology 141, 367–373. 19836905 10.1016/j.vetmic.2009.09.021

[vms381-bib-0025] Paton D. , McGoldrick A. , Greiser‐Wilke I. , Parchariyanon S. , Song J.‐Y. , Liou P. *et al* (2000) Genetic typing of classical swine fever virus. Veterinary microbiology 73, 137–157. 10785324 10.1016/s0378-1135(00)00141-3

[vms381-bib-0026] Postel A. , Moennig V. & Becher P. (2012) Classical swine fever in Europe–the current situation. Berliner und Munchener tierarztliche Wochenschrift 126, 468–475. 24511821

[vms381-bib-0027] Prasad K.N. , Verma A. , Srivastava S. , Gupta R.K. , Pandey C.M. & Paliwal V.K. (2011) An epidemiological study of asymptomatic neurocysticercosis in a pig farming community in northern India. Transactions of the Royal Society of Tropical Medicine and Hygiene 105, 531–536. 21764415 10.1016/j.trstmh.2011.06.001

[vms381-bib-0028] Ravishankar C. , Priya P. , Mini M. , Rameshkumar P. , Selvan P.S. , Jayesh V. *et al* (2007) First confirmed occurrence of classical swine fever in Kerala state, India. Journal of swine health and production 15, 156.

[vms381-bib-0029] Sarma D. , Krishna L. & Mishri J. (2008) Classical swine fever in pigs and its status in India: A review. The Indian Journal of Animal Sciences 78, 12.

[vms381-bib-0030] Sarma D.K. , Mishra N. , Vilcek S. , Rajukumar K. , Behera S.P. , Nema R.K. *et al* (2011) Phylogenetic analysis of recent classical swine fever virus (CSFV) isolates from Assam, India. Comparative immunology, microbiology and infectious diseases 34, 11–15. 10.1016/j.cimid.2009.09.005 19896713

[vms381-bib-0031] Song J.Y. , Lim S. , Jeoung H. , Choi E.J. , Hyun B.H. , Kim B. *et al* (2013) Prevalence of classical swine fever virus in domestic pigs in South Korea: 1999–2011. Transboundary and Emerging Diseases 60, 546–551. 22925439 10.1111/j.1865-1682.2012.01371.x

[vms381-bib-0032] van Oirschot J. (2003) Vaccinology of classical swine fever: From lab to field. Veterinary microbiology 96, 367–384. 14599784 10.1016/j.vetmic.2003.09.008

